# Autism Information Progression and the Impact of Misinformation on Autism Knowledge, Awareness and Stigmatization

**DOI:** 10.3390/bs16050660

**Published:** 2026-04-27

**Authors:** Nayana Pampapura Madali, Suliman Hawamdeh

**Affiliations:** 1 School of Professional Studies, University of Kansas, Overland Park, KS 66213, USA; 2 Department of Information Science, University of North Texas, Denton, TX 76203-501, USA; suliman.hawamdeh@unt.edu

**Keywords:** autism, knowledge, awareness, stigma, misinformation

## Abstract

Recent studies have shown a growing prevalence of autism spectrum disorders, accompanied by heightened concerns about the impact of misinformation on autism stigmatization, shaping public perceptions of autism. With the increase in autism cases worldwide, it is critical to have sufficient understanding, knowledge, and awareness about autism, especially among the autism information seekers. This study focused on the progression of autism information over time and investigated the relationships among various factors such as autism knowledge, awareness, stigma, misinformation, cultural beliefs, and social norms. Employing a two-phase research design approach comprising systematic literature review and survey, the study indicated an overall increase in autism knowledge and awareness, although it revealed disparities in certain ethnicities and areas such as genetic testing. Despite advancements, stigma was found to persist. Survey findings validated these observations, emphasizing the necessity for heightened autism awareness and the continued presence of stigma. Furthermore, the survey demonstrated that knowledge influences awareness, whereas cultural beliefs and social norms directly affect autism misinformation. Importantly, the study highlighted how cultural beliefs, and misinformation can hinder accurate understanding and knowledge of autism, potentially exacerbating stigma. By employing evidence-based approaches, this study offers comprehensive insights into autism, enriching the broader literature on the subject.

## 1. Introduction

Knowledge, perceptions, and attitudes toward medical conditions evolve over time as research advances and information becomes more widely disseminated. Understanding these changes is important because they shape public awareness, influence decision-making, and affect the experiences of patients, families, and caregivers. Although research on autism knowledge, awareness, and stigma has grown substantially over the past two decades, most studies examine these constructs independently or within specific populations (Heidgerken et al., 2005; Dillenburger et al., 2017; Turnock et al., 2022). Consequently, limited research has explored how these factors interact or how they have evolved collectively over time, especially in the context of autistic individuals, family members or caregivers. In particular, the role of sociocultural influences and the increasing spread of online misinformation in shaping autism knowledge and awareness remains underexamined. Addressing this gap is important for understanding how autism-related information is interpreted by autistic individuals and their families or caregivers and for identifying factors that may contribute to misconceptions or stigma.

Autism spectrum disorder (ASD) is a neurodevelopmental condition characterized by differences in social communication, interaction, and patterns of restricted or repetitive behaviors (WHO, 2023; American Psychological Association, 2013). Autism is a highly heterogeneous spectrum characterized by substantial variation in communication abilities, cognitive functioning, adaptive skills, and support needs. It is also important to recognize that a substantial proportion of autistic individuals have co-occurring intellectual disabilities, although this group has often been underrepresented in autism research. Over time, the understanding of autism has evolved significantly, from being considered a form of schizophrenia to recognition as a spectrum disorder with complex genetic and environmental determinants (Salari et al., 2022). With increasing global prevalence, it is essential for individuals seeking autism-related information to have accurate knowledge and awareness, and to recognize associated stigma (Zeidan et al., 2022; Salari et al., 2022).

Autism knowledge refers to the factual understanding of autism characteristics, causes, and treatments, whereas autism awareness refers to the recognition and general familiarity with autism and its societal implications. Over the past few decades, autism knowledge and awareness have been the focus of numerous studies (Dillenburger et al., 2017; Altay, 2019). Research indicates that knowledge about autism varies across populations, with parents, educators, and health professionals often demonstrating gaps in understanding (Heys et al., 2017; Hussein et al., 2019). Research also indicates that autistic individuals with sufficient language ability tend to have higher knowledge and lower stigma compared to non-autistic populations (Gillespie-Lynch et al., 2017). Although increased awareness is often assumed to reduce stigma, prior research suggests that awareness alone may be insufficient to reduce stigmatizing attitudes when stereotypes, emotional biases, or deeply entrenched beliefs persist (Shim et al., 2022). Awareness may increase recognition of autism without necessarily transforming evaluative judgments or behavioral intentions.

Stigma is defined as a social process of exclusion, rejection, or devaluation, often arising from misconceptions or negative attitudes (Weiss et al., 2006). Autism stigma refers to the negative attitudes toward autistic individuals and autism has historically been associated with stigma due to misconceptions about causes and behaviors, and such stigma can negatively affect the autistic individuals, their family members and their caregivers (Papadopoulos et al., 2019; Turnock et al., 2022). Cultural beliefs and social norms also have affected autism and research is carried out in this area as well. Research shows that cultural beliefs and community attitudes strongly influence how autism is perceived and treated. Stigma, misconceptions about causes (e.g., genetics, environmental factors, or supernatural beliefs), and cultural norms can affect families’ treatment decisions and contribute to higher levels of autism stigma in certain communities (Kang-Yi et al., 2018; Hebert & Koulouglioti, 2010; Alqahtani, 2012; Papadopoulos, 2016; Kim et al., 2022).

The growth of social media and the internet have made it easier to disseminate misinformation. As more people turn to these resources for health information, incorrect information about health-related concerns continues to be spreading (Krishna & Thompson, 2021). There is a lot of misinformation about autism as well (Keenan & Dillenburger, 2018). Autism misinformation refers to the inaccurate or misleading information about autism, often spread through media or social networks. Misinformation about autism, such as myths linking vaccines to autism, assumptions that all autistic individuals have mental health problems, or beliefs that all autistic people share the same cognitive abilities, continues to spread through social and traditional media, contributing to misunderstanding and stigma (Krishna & Thompson, 2021; White, 2014).

From 2000 to approximately 2010, public understanding of autism was comparatively limited, and misconceptions were widespread. Autism was often poorly differentiated from other developmental or psychiatric conditions, contributing to stigma and delayed recognition (Grinker, 2020; Yu et al., 2020). Information access was more restricted, and awareness efforts were less visible. Between 2010 and 2020, autism awareness expanded substantially through advocacy, research growth, and broader diagnostic recognition. A major milestone was the shift toward understanding autism as a spectrum disorder, emphasizing heterogeneity rather than a narrow or singular presentation (American Psychological Association, 2013). This period saw increased public visibility and improved recognition, although disparities across racial, ethnic, and socioeconomic groups remained. From 2020 to 2025, autism information became more accessible through digital platforms and social media (Jawed et al., 2023). However, increased access also coincided with the spread of misinformation, conflicting narratives, and uneven information quality (Skafle et al., 2024; Keenan & Dillenburger, 2018). Genetic testing practices related to autism also changed during this broader period. Earlier studies often reflected more limited testing options and lower accessibility, whereas more recent clinical practice increasingly includes broader genomic approaches such as exome sequencing and whole-genome sequencing for selected neurodevelopmental presentations (Selvanayagam et al., 2025; Lowther et al., 2023; Trost et al., 2022). Overall, the 2000–2025 period reflects a transition from limited access to autism-related information to widespread information availability, alongside major advances in autism understanding, while persistent sociocultural and structural barriers continue to shape how autism is perceived across communities.

Considering factors such as knowledge, awareness, stigma, cultural beliefs, social norms, and misinformation, it is important to examine how these elements shape perceptions of autism, particularly among autistic individuals, parents, and caregivers. Despite advances in autism research and public awareness initiatives, gaps remain in understanding how autism knowledge, awareness, and stigma have progressed over time, particularly in relation to the impact of misinformation and cultural beliefs. This study addresses these research gaps by examining the progression of autism information, knowledge, awareness, and stigma over time, as well as the influence of misinformation, cultural beliefs, and social norms especially among autistic individuals, parents, and caregivers.

The Knowledge-Attitude-Behavior (KAB) model, widely used in health education (Xu et al., 2010), explains the relationship between knowledge, attitude, and behavior. In this model, knowledge refers to what a person knows, attitude to what they think, and behavior to how they act. The model suggests that knowledge influences attitude, which in turn affects behavior. While extensively applied in various research fields (Zwilling et al., 2022; Iyer, 2018; Colson et al., 2014; Albaqawi et al., 2020), it has been rarely applied to autism studies to investigate how knowledge affects awareness and stigmatization behavior.

The KAB model provides a useful heuristic framework for examining possible relationships among knowledge, attitudes, and behavioral outcomes. However, critics note that human behavior is not always linear or purely rational, and social, emotional, and structural factors may also shape outcomes. Additionally, in the modern information landscape, this linear progression from knowledge to attitude to behavior might be disrupted, particularly by the influence of digital media. Misinformation frameworks related to health suggest that exposure to misinformation or misleading information can distort knowledge, which in turn can reinforce misconceptions and thus can affect the attitudes that might not align with scientific evidence (Krishna & Thompson, 2021). Similarly, cultural beliefs and social norms provide a broader contextual framework that can shape how autism-related information is interpreted, accepted, or resisted. Additionally, stigma theory conceptualizes that stigma is a socially constructed process that is driven by labeling, stereotyping, and social exclusion, which is often reinforced through cultural narratives and misinformation (Weiss et al., 2006; Papadopoulos et al., 2019). This study extends KAB model by integrating all these perspectives to better capture the complexity of autism-related information processing. In this study, autism awareness is conceptualized as an attitudinal orientation reflecting openness, recognition, and sensitivity toward autism-related issues. Although awareness is not identical to attitude, it captures evaluative and affective readiness that may precede behavior. Similarly, stigmatization is modeled as a behavioral manifestation of negative attitudes, expressed through social distancing, discriminatory intentions, or exclusionary judgments. By adding additional constructs such as misinformation, cultural beliefs and social norms to the KAB model, this study tries to examine how these external influences may shape or disrupt the relationship between autism knowledge, and awareness. In this extended framework, knowledge does not operate individually but is refined through sociocultural contexts and information ecosystems, which may boost or weaken its impact on awareness and stigma. This approach allows for a better understanding about how autism-related perceptions are formed and how misinformation and cultural beliefs and social norms impact autism knowledge and awareness.

Using the Knowledge–Attitude–Behavior (KAB) model as a conceptual framework, this study examines the relationships among autism knowledge, awareness, and stigmatization. The KAB model was adapted to align with the variables examined in this study. Specifically, knowledge represents autism knowledge, attitude corresponds to autism awareness, and behavior reflects autism stigmatization. In addition, the framework was extended to examine the influence of cultural beliefs, social norms, and misinformation on autism knowledge and awareness.

Below are the study’s research questions:

RQ1: How has information about autism evolved over time, and what is the state of autism knowledge, awareness, and stigmatization in recent years?
RQ2: What is the impact of autism knowledge on autism awareness and stigmatization behavior?
RQ3: What is the impact of misinformation on autism knowledge and autism awareness?
RQ4: What is the impact of cultural beliefs and social norms on autism knowledge, awareness, and misinformation?

By exploring these questions, this study provides a comprehensive overview of autism knowledge, awareness, and stigma progression, offering insights for healthcare professionals, researchers, autistic individuals, and their families.

## 2. Methodology

**Figure 1:**
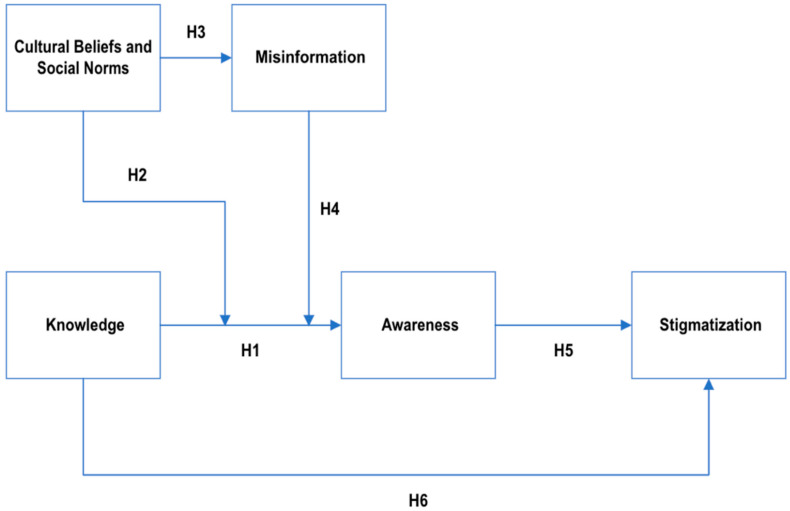
Conceptual model.

In this study, the KAB model is used to develop a conceptual framework to understand the impact of autism knowledge on awareness and stigmatization behavior. The conceptual model also explores the relationship between cultural beliefs, social norms, and misinformation about autism, highlighting the interconnectedness of these constructs. Figure 1 depicts the conceptual framework for the current study.

This study formulated and tested six hypotheses based on the proposed conceptual model, which are presented below.

Knowledge, defined as understanding gained through education or experience, plays an important role in shaping attitudes toward autism. Additionally, health literacy theory supports the relationship between knowledge and awareness, which proposes that individuals’ ability to access, understand, and interpret health information directly influences their awareness and decision-making processes (Sørensen et al., 2012). Research shows that knowledge about autism symptoms, diagnosis, and treatment helps dispel misconceptions and improve attitudes, thereby increasing autism awareness (Kuzminski et al., 2019; Lu et al., 2020). Also from a cognitive processing perspective, enhanced knowledge lessens ambiguity and uncertainty, thus allowing the individuals to form more accurate mental representations of autism. This aligns with dual-process theories of cognition, which suggest that knowledgeable individuals are more likely to engage in deliberate, analytical thinking rather than relying on cognitive shortcuts, leading to greater awareness and understanding (Brosnan et al., 2016). So, it is hypothesized the following:

**H1.**  *Autism knowledge is positively correlated with autism awareness.*

Social norms and cultural beliefs are shared ideas and unwritten rules passed down through generations that influence how people think and behave. Although these beliefs may not always be supported by scientific evidence, they are often accepted without question and can significantly shape individuals’ knowledge and attitudes (Yoo et al., 2019; Jegede & Okebukola, 1991; Sheikh & Furnham, 2000). Research shows that such beliefs and norms can contribute to the development of stigma, particularly regarding medical and mental health conditions (Quinn & Knifton, 2014; Ran et al., 2021). They may also facilitate the spread of misinformation, as culturally rooted ideas can make false information appear more credible (Gupta et al., 2023). These insights led to the development of the following hypotheses.

**H2.**  *Cultural beliefs and social norms have direct correlations to autism knowledge and awareness.*

**H3.**  *There is a direct relationship between cultural beliefs, social norms, and autism misinformation.*

Misinformation refers to inaccurate information that is often created or shared in ways that mislead people. In the healthcare context, misinformation can lead to serious consequences such as delayed diagnosis, inappropriate treatment, or broader public health risks (Au et al., 2021). Information disorder theory explains how misinformation spreads within digital environments easily and this in turn shapes the public’s understanding (Wardle & Derakhshan, 2017). This theory highlights how misinformation spreads quicker than actual or accurate information. In the context of autism, misinformation can distort autism knowledge and awareness and interfere with the KAB pathway. Increasing awareness of misinformation is therefore important for identifying myths, promoting reliable information sources, and reducing the spread of false information. In the context of autism, awareness of misinformation can help individuals recognize inaccurate claims, encourage the dissemination of accurate information, and ultimately improve autism knowledge and awareness. The relationship between autism misinformation and knowledge is also more complex than a simple inverse association. This relationship may depend on whether knowledge is conceptualized as objective factual accuracy, self-perceived familiarity, or general exposure to autism-related information. Individuals with greater exposure to autism content may acquire both accurate information and misinformation simultaneously. Accordingly, the present study examines these relationships empirically rather than assuming that misinformation is always negatively associated with knowledge. Thus, it is hypothesized that:

**H4.**  *Misinformation has a direct correlation to autism knowledge and autism awareness.*

Stigma is a social phenomenon driven by labeling and negative attitudes that tend to separate and devalue groups, thus resulting in structural and interpersonal discrimination. Autism is highly stigmatized in many societies, leading to negative consequences such as social isolation, discrimination, and mental health challenges for autistic individuals and their families (Tang & Bie, 2016; Papadopoulos, 2016). Stigma theory provides the grounding for the relationship between stigma and awareness (Goffman, 2009). According to the stigma theory, increased awareness might reduce the stigma by challenging stereotypes and promoting more accurate understandings of autism. In addition, contact theory suggests that familiarity with a stigmatized group acts as an influential mechanism for reducing prejudice by lowering fear, anxiety and uncertainty (Pettigrew & Tropp, 2008). Awareness can act as a form of indirect contact, especially when people are exposed to accurate representations of autism. Increasing autism awareness helps people access reliable information, reject misconceptions, and better understand the condition, which can reduce stigmatizing attitudes and behaviors. Hence, it is hypothesized that:

**H5.**  *Autism awareness negatively correlates with autism stigmatization.*

**H6.**  *Autism awareness serves as a mediator between knowledge and stigmatization.*

To examine these hypotheses and explore the progression of autism knowledge, awareness, and stigma, this study adopted a two-phase research design consisting of a systematic review and a survey. The first phase involved a systematic literature review following the Preferred Reporting Items for Systematic Reviews and Meta-Analyses (PRISMA) 2020 guidelines (PRISMA, n.d.) to provide an overview of how autism knowledge, awareness, and stigma have evolved over time and to examine the relationships among these variables. The second phase involved a survey to validate the review findings, assess the impact of autism knowledge on awareness and stigma using the KAB model, examine the influence of cultural beliefs, social norms, and misinformation and test the study’s hypothesis.

## 3. Data Collection and Analysis

### 3.1. Phase 1: Systematic Review

In phase 1, a systematic literature review was conducted to address RQ1 regarding the progression of autism knowledge, awareness, and stigma. The review followed the PRISMA 2020 guidelines, checklist and flowchart (PRISMA, n.d.) to ensure transparency and replicability. Journal articles related to autism knowledge, awareness, and stigma were collected and analyzed to understand the progress of autism information over the years. Journal articles published between 2000 and 2025 were included in the review. This timeframe was selected because it reflects a period of substantial development in the scientific understanding of autism (Happé & Frith, 2020) and allows for the inclusion of a sufficiently broad body of literature.

For the systematic literature review, the inclusion and exclusion criteria were defined first, based on which the search was conducted, and relevant records or sources were retrieved.

The inclusion criteria are shown below:

- Study should be related to autism knowledge, awareness, or stigma.
- Study should be in English.
- The study participants should be either autistic individuals or family members. The review focused on autistic individuals and their families because these groups are directly affected by autism-related knowledge, awareness, and stigma.
- The study participants should be located in the U.S. Study selection was restricted to U.S. residents to ensure alignment with Phase 2 of the study, which involved a survey conducted exclusively among participants in the United States. While this criterion reduced the breadth of the evidence base, it improved contextual consistency between the review and survey phases.

The exclusion criteria are:

- Studies published before 2000 and after 2025 were excluded.
- The study should not be a review or an opinion article.

Three databases—PubMed, Web of Science, and Scopus—were queried using keywords related to autism, knowledge, awareness, stigma, and participants (autistic individuals, family, caregivers). Below table lists the research question and the search terms (Table 1).

**Figure 2:**
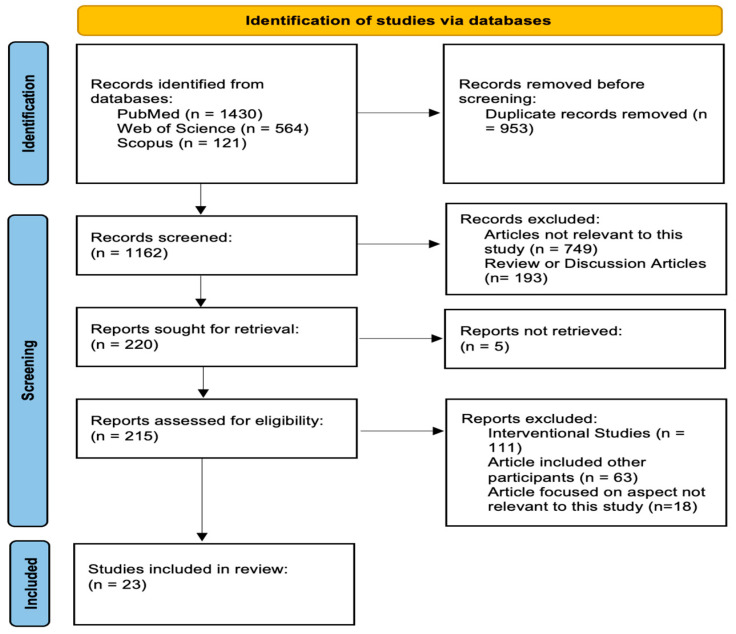
PRISMA flow diagram.

Initially, 2115 articles were identified, and 1162 duplicates were removed. After applying inclusion and exclusion criteria, 215 articles were screened. Among these, 111 interventional studies were excluded because the study’s review focused specifically on perceptions and understanding of autism information rather than evaluating intervention outcomes. This resulted in identifying 23 relevant studies focusing on autism knowledge, awareness, or stigma. Figure 2 presents the PRISMA flow diagram, illustrating the step-by-step process of study identification, screening, eligibility assessment, and inclusion in the review.

Data pertaining to the 23 journal articles was extracted using a standardized form capturing study characteristics, participant details, outcomes, and key findings. A thematic analysis was conducted on the finalized articles to ensure they met the predefined inclusion and exclusion criteria and aligned with the scope of the study. This process helped confirm the relevance and suitability of the selected literature for addressing the research question.

To ensure reliability, a team of two experts reviewed the article selection to minimize bias, ensure inclusion quality, and assess the strength of the evidence, with any disagreements resolved through discussion. Internal validity was also used to reduce selection bias in the systematic literature review. Additionally, after coding the full texts, the codes were reviewed by a third person to confirm their consistency and reliability.

After finalizing the 23 included journal articles, a structured qualitative content analysis, including conceptual analysis, was conducted to examine the progression of autism knowledge, awareness, and stigma over time. NVivo 14 and Microsoft Excel were used to code and categorize the literature, with Excel summarizing each article and its contributions to understanding the progression of autism-related information.

The strength and certainty of the studies were evaluated using the GRADE framework, and the findings were synthesized to identify the progression of autism knowledge, awareness, and stigma. Each study was evaluated based on research design, sample size, data collection methods, and potential sources of bias. Studies employing large samples and robust designs (e.g., surveys with validated instruments) were rated as high quality, while cross-sectional and self-reported studies were generally classified as moderate. Qualitative studies with small sample sizes or limited generalizability were rated as low quality. Quality appraisal of the included studies showed mixed methodological strength. Most studies were rated as moderate quality, and these studies were generally survey-based and benefited from comparatively larger sample sizes. Across the reviewed literature, the most frequent methodological limitations involved small sample sizes, convenience or purposive sampling approaches, reliance on self-reported data, and predominantly cross-sectional or descriptive designs. Overall, the certainty of evidence was considered low to moderate, reflecting variation in study designs, data collection methods, and methodological rigor across the included studies. The results of the quality assessment are presented in Table 2.

### 3.2. Phase 2: Survey

In Phase 2, a survey was used to collect data on autism knowledge, awareness, stigma, cultural beliefs, social norms, and misinformation, as outlined in the conceptual model. The survey aimed to examine the relationships among these variables as well as validate findings from the systematic literature review.

Some survey questions were adapted from the Autism Stigma and Knowledge Questionnaire (ASK-Q) (Harrison et al., 2017), a validated tool assessing autism knowledge across four subscales: symptoms/diagnosis, etiology, treatment, and stigma, which has been used across diverse populations, including parents, healthcare professionals, and college students (Handayani & Paramita, 2020; Reckard, 2021). Only items relevant to this study were adopted and measured on a five-point Likert scale to capture gradations in agreement or endorsement rather than dichotomous responses. This approach was intended to increase response variability and support correlational and regression-based analyses. However, modifying the original response format may alter the psychometric properties of the instrument, and findings should therefore be interpreted cautiously.

Additional survey questions/items were developed based on literature review, the conceptual model, and expert input to assess autism awareness, cultural beliefs, social norms, and misinformation. These items were reviewed for content validity by an expert, and reliability for the full survey. The 31-question survey, administered via Qualtrics, included both open- and closed-ended items and took approximately 30 min to complete.

Before administering the main survey, a pilot study was conducted to assess validity and reliability. Survey items, including adapted ASK-Q items and newly developed items assessing awareness, cultural beliefs, social norms, and misinformation, underwent content analysis and expert review to ensure they measured the intended constructs without bias. Internal consistency reliability was assessed using Cronbach’s alpha (Tavakol & Dennick, 2011), as an initial evaluation of scale performance. Exploratory or confirmatory factor analysis was considered; however, given the modest sample size relative to the number of measured items and the exploratory nature of the study, full latent-variable validation was not considered statistically stable in the present dataset. Accordingly, reliability estimates are presented as preliminary evidence, and further validation with larger samples is recommended.

The main survey employed purposive sampling, a non-probability method in which participants are selected based on predefined criteria (Jordan, 2021; Renjith et al., 2021). Autistic individuals, family members, and caregivers were chosen as participants, informed by the systematic literature review, which primarily included studies involving these groups. This alignment allowed the study to examine autism knowledge, awareness, and stigma from their perspectives, as well as relationships among autism-related variables. Participants were recruited via Amazon Mechanical Turk (MTurk) and the University of North Texas (UNT) Kristin Farmer Autism Center, targeting U.S. residents. To collect responses from MTurk, a project was created and a Qualtrics survey link was included in the project. MTurk recruitment criteria included U.S. residency, a minimum of 5000 completed HITs, a ≥90% approval rate, and eligibility limited to individuals with autism or their family members. Additionally, a study flyer with the survey link was distributed through the autism center to relevant participants. The survey questionnaires (Supplementary Materials) were completed entirely online using Qualtrics. Survey participants received $1 in compensation, either credited directly to their Amazon Mechanical Turk account or distributed via Tango Card. A total of 178 responses were collected, of which 159 were deemed suitable for analysis, representing a diverse sample in terms of background, gender, race, and ethnicity.

Survey responses were analyzed using IBM SPSS Statistics 29 to examine relationships among autism variables. Given the cross-sectional and exploratory nature of the study, as well as sample size considerations, the analytic strategy focused on a stepwise approach using descriptive statistics, correlations, multiple regression, and mediation analysis. This approach allowed for the examination of individual and indirect associations among variables without imposing assumptions of full latent variable structure. NVivo was used for qualitative analysis, including word cloud visualization of open-ended responses (Handayani & Paramita, 2020; Reckard, 2021).

## 4. Results

This study adopted a two-phase approach to address the research questions. In the first phase, a systematic literature review was conducted following PRISMA 2020 guidelines to examine the progression of autism knowledge, awareness, and stigma among autistic individuals and their families and caregivers (PRISMA, n.d.). The review searched three databases—PubMed, Web of Science, and Scopus, which resulted in the identification of 23 relevant studies on autism knowledge, awareness, or stigma.

Five of the twenty-three studies examined autism knowledge among autistic individuals or family members. Participant numbers ranged from 16 to 99, with interviews being the most used research instrument. The findings indicated varying levels of autism knowledge across different domains. For example, families demonstrated adequate knowledge of research-based early intervention services (Sansosti et al., 2012), whereas parents reported limited awareness of genetic testing for autism spectrum disorders (Hanish et al., 2018). Additionally, three studies compared autism knowledge across ethnic groups and found disparities in knowledge levels. While White mothers generally demonstrated greater knowledge of ASD, Latina mothers showed lower awareness, particularly regarding developmental milestones. Although knowledge increased following a child’s ASD diagnosis, Latina mothers reported difficulties accepting the diagnosis and applying their knowledge to understand their child’s needs (Ratto et al., 2016; Burke et al., 2018; Gordillo et al., 2020). These findings highlight the influence of cultural factors on autism knowledge.

Nine of the twenty-three studies focused on autism awareness among autistic individuals and family members. Participant numbers ranged from 22 to 155, and interviews and surveys were the primary research methods. These studies were categorized into three areas: genetic testing, awareness across ethnicities, and treatment awareness. Four studies examining genetic testing reported insufficient awareness due to factors such as limited knowledge and financial constraints (Vande Wydeven et al., 2012; Chen et al., 2013; Harrington et al., 2018; Zebolsky et al., 2020). Three studies addressing ethnic differences found no significant disparities in the age of ASD diagnosis between Hispanic and non-Hispanic White children, suggesting improvements in awareness and access to support (Magana et al., 2013; Ijalba, 2016; Ferguson & Vigil, 2019). Moreover, these studies show that there has been an increase in autism awareness over the years among certain ethnicities yet there is a need for autism awareness among other ethnicities. Two studies related to treatment indicated that while parents were generally aware of autism treatments, certain ethnic communities had limited knowledge (Deyro et al., 2016; Rosales et al., 2021). Overall, these findings highlight the need to increase awareness of genetic testing, improve treatment knowledge, and address awareness gaps across ethnic communities.

There were nine studies identified related to autism stigma and these articles examined autism stigma among autistic individuals and their families. Interviews were the most used research method, and despite differences in sample sizes and study periods (2014–2022), all studies reported the persistence of autism stigma. One study emphasized the need to reduce societal stigma associated with ASD and disability labels (Jones et al., 2015). Other studies reported that Latino communities often face limited access to ASD-related information and experience stigma related to mental health and disabilities (Zuckerman et al., 2014, 2018; Blanche et al., 2015; Cohen & Miguel, 2018; Dababnah et al., 2018; Habayeb et al., 2020; Stein Duker et al., 2022). Misunderstandings about ASD were also found to contribute to social isolation (Marsack & Perry, 2018).

**Figure 3:**
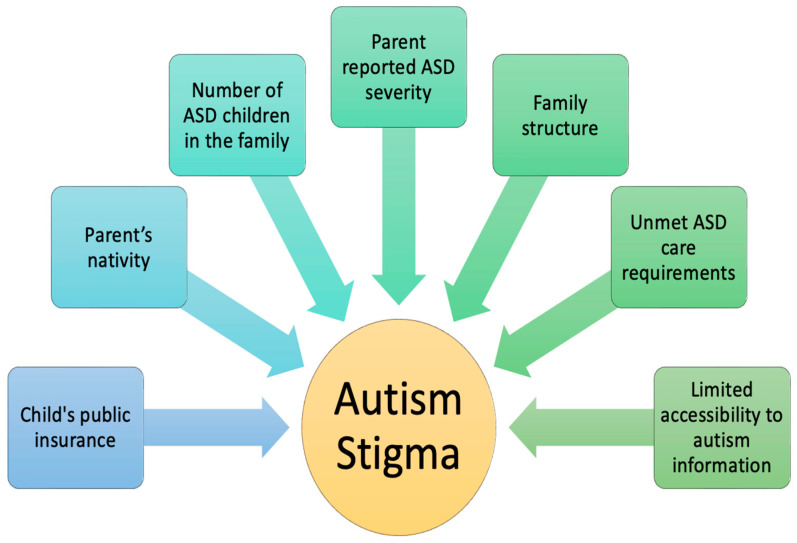
Factors that impact autism stigma.

The review also identified several factors associated with autism stigma, including child public insurance status, parental nativity, the number of children with ASD in a household, parent-reported severity of ASD, family structure, unmet care needs, and limited access to autism information (Zuckerman et al., 2018; Stein Duker et al., 2022), as illustrated in Figure 3. These factors likely relate to stigma through multiple pathways, including caregiving stress, perceived support burden, service barriers, and broader social disadvantage, rather than through knowledge deficits alone.

Overall, the systematic literature review examined the evolution of autism-related information, focusing on knowledge, awareness, and stigma. Although progress has been made in increasing autism knowledge and awareness, gaps remain in areas such as genetic testing and within certain ethnic communities. The findings also highlight the persistent presence of autism stigma, suggesting that it is a complex issue influenced by multiple factors beyond knowledge and awareness, including social norms, caregiving experiences, and support needs. While educational and awareness initiatives may contribute to improved understanding, they are unlikely to address all stigma-related factors on their own, and broader, multi-level approaches that also respond to family needs, service access, and social contexts may be needed.

In Phase 2, a survey was conducted among autistic individuals, family members, and caregivers to assess perceptions of autism knowledge, awareness, and stigma and to examine correlations among autism-related variables. A pilot study with six responses was first conducted to assess survey reliability using Cronbach’s alpha. Each autism variable consisted of multiple items, and the mean values of these items were used to calculate reliability. As shown in Table 3, the variables demonstrated varying levels of internal consistency, with autism stigma showing acceptable reliability and autism misinformation demonstrating the highest reliability.

Following the pilot study, the survey was distributed via Amazon Mechanical Turk and the UNT Kristin Farmer Autism Center. Participants first completed a consent agreement before proceeding with the 31 survey questions. The questionnaire was organized into seven sections: (1) Demographics (3 questions), (2) Background (7 questions examining participants’ relationship with and understanding of autism), (3) Autism Knowledge (4 questions), (4) Autism Awareness (4 questions), (5) Autism Stigma (4 questions), (6) Autism Cultural Beliefs and Social Norms (4 questions), and (7) Autism Misinformation (5 questions examining misconceptions and sources of misinformation).

A total of 177 responses were collected, of which 18 were excluded due to incomplete or irrelevant responses, resulting in 159 valid responses for analysis. Although the sample size is modest, it is comparable to exploratory studies examining perceptions of autism within specific populations. Standard deviations (SD) ranged between 0.45 and 0.72 from their respective means, providing additional insights into the variability of the data points. As shown on the table below (Table 4).

The combined data from both platforms were analyzed using NVivo and IBM SPSS. Table 5 presents the demographic characteristics of the participants, indicating that most respondents were male, primarily between the ages of 18 and 30, and predominantly identified as White.

Survey responses related to background (Table 6) showed that all participants had prior knowledge of autism, either as autistic individuals or family members. Personal experience was the primary source of information, followed by doctors and medical professionals. Secondary sources additionally included the internet and educational institutions, highlighting the role of online resources and academic settings in disseminating autism-related information.

The survey also included questions to understand participants’ perceptions and understanding of autism-related knowledge, awareness, and stigma. The collected responses were analyzed to evaluate the levels of autism knowledge, awareness, and stigma among participants. The results indicated that most respondents demonstrated a good understanding of autism. However, the analysis also revealed discrepancies in autism awareness, suggesting that participants possessed varying levels of awareness. Additionally, the findings indicated the presence of some level of autism-related stigma among the surveyed participants.

### 4.1. Hypothesis Testing

The study used IBM SPSS to conduct a correlation analysis to examine the relationships among various autism variables, such as knowledge, awareness, stigma, cultural beliefs, social norms, and misinformation using the survey data. Pearson correlation analysis was used to determine the strength of the linear relationship between the variables. Multiple-choice items were analyzed to assess specific aspects of autism, and the resulting mean value was used to calculate the Pearson correlation and test the study hypothesis.

**H1.**  *Autism knowledge is positively correlated to autism awareness.*

The first hypothesis posits a positive correlation between autism knowledge and awareness, based on the idea that gaining knowledge about autism increases awareness. A Pearson correlation analysis of the variables showed a correlation coefficient of 0.455, with a significance level of less than 0.001, supporting the hypothesis. This indicates that higher autism knowledge is associated with increased awareness.

**H2.**  *Cultural beliefs and social norms have direct correlations to autism knowledge and awareness.*

The second hypothesis suggests that cultural beliefs and social norms influence autism knowledge and awareness. The study found a significant positive correlation between cultural beliefs and social norms and autism knowledge, with a Pearson correlation coefficient of 0.381 and a significance level of less than 0.001. However, no statistically significant association was found between cultural beliefs and social norms and autism awareness (r = 0.094, *p* = 0.118). Therefore, hypothesis 2 was partially supported, indicating a significant correlation between cultural beliefs and social norms and autism knowledge.

**H3.**  *There is a direct relationship between cultural beliefs, social norms, and autism misinformation.*

The hypothesis suggests that cultural beliefs and social norms are linked to autism misinformation. This hypothesis was formulated based on the premise that individuals who hold stronger cultural beliefs are more likely to endorse stigma and misinformation regarding autism, resulting in misconceptions about the condition. A correlation analysis showed a positive correlation coefficient of 0.605 with a significance level of less than 0.001, confirming hypothesis three and suggesting that stronger cultural beliefs are linked to higher endorsement of misinformation.

**H4.**  *Misinformation has a direct correlation to autism knowledge and autism awareness.*

The fourth hypothesis posits a direct link between autism misinformation, knowledge, and awareness, grounded on the idea that misinformation obscures understanding and awareness of autism. However, a correlation analysis shows a positive correlation between misinformation and knowledge (correlation coefficient of 0.386, significance of <0.001), but there is no significant relationship between misinformation and autism awareness (correlation coefficient of 0.073, significance of 0.180). Therefore, hypothesis 4 was not fully validated.

**H5.**  *Autism awareness negatively correlates with autism stigmatization.*

The fifth hypothesis, assuming increased autism awareness would reduce stigma. However, Pearson correlation analysis indicated a statistically significant positive relationship between awareness and stigma (r = 0.166, *p* = 0.018). Because the direction of the observed relationship contradicted the hypothesized negative association, H5 was not supported.

**H6.**  *Autism awareness serves as a mediator between knowledge and stigmatization.*

The sixth hypothesis suggests that increased autism knowledge leads to greater awareness, which reduces autism stigma. This hypothesis was tested using Andrew F. Hayes’ mediation process analysis, which examines the relationship between two variables with the introduction of a third hypothetical variable as a mediator. Autism knowledge was the independent variable, and autism stigma was the dependent variable with autism awareness being the mediator variable. The outcome model indicated a significant positive direct effect of knowledge on stigma (B = 0.492, *p* < 0.001). However, awareness did not significantly predict stigma when controlling for knowledge (B = −0.027, *p* = 0.809). Based on the available mediation output, no evidence was found to support awareness as a significant mediator. Below Table illustrates Andrew F. Hayes mediation process analysis (Table 7).

Overall, the hypothesis testing outcomes revealed two confirmed, two partially supported, and two rejected hypotheses. The results highlight the relationship between cultural beliefs and social norms with knowledge of autism, as well as the impact of misinformation on knowledge.

### 4.2. Research Questions

A systematic literature review was conducted to examine the progression of autism knowledge, awareness, and stigma (RQ1). Five articles revealed varying levels of knowledge across autism aspects. White mothers showed superior knowledge compared to Latina mothers, indicating cultural differences. The review highlighted the need for enhanced understanding, particularly in specialized areas like genetic testing. Families also had knowledge about research-based interventions. Despite progress, there is still a need for bolstering autism knowledge within specific communities and facets like genetic testing. Nine articles on autism awareness showed a significant gap in genetic testing awareness due to financial constraints and limited access. While autism awareness has increased in some ethnic groups, greater awareness is still needed across all ethnicities. Awareness of treatments was generally sufficient but deficient in certain ethnic communities. The review also identified nine articles on autism stigma, highlighting its persistence, especially within the Latino community, where access to ASD information and stigma related to mental health and disabilities were issues. Misconceptions about ASD contributed to social isolation.

The review stresses the need for improved autism knowledge and awareness, particularly in genetic testing, supported by studies like Li et al. (2016). It advocates healthcare providers and genetic counselors to be well-educated to meet the needs of parents and caregivers. Health education programs can enhance communication and awareness. Additionally, the review highlights insufficient knowledge of autism within specific ethnic groups, as shown by studies from Colbert et al. (2017) and Lopez et al. (2018). Educational programs tailored to diverse communities, along with culturally sensitive interventions, are needed. Media platforms and parent-professional collaboration can also aid in disseminating knowledge. Finally, the review underscores the persistence of autism stigma, supported by studies like Papoudi et al. (2021), Liao et al. (2019) and Salleh et al. (2020). Awareness campaigns and educational initiatives are crucial to combating stigma. Educating healthcare professionals about the challenges parents face and fostering supportive environments are also key to reducing stigma and supporting families with ASD.

To validate these findings, a survey of 159 autistic individuals and family members assessed perceptions of knowledge, awareness, and stigma. Results showed that participants generally possessed satisfactory knowledge of autism, although awareness and stigma varied. Overall, the combined findings indicate that while knowledge and awareness have progressed, disparities and persistent stigma remain key areas for intervention, providing a comprehensive picture of the current state and evolution of autism-related understanding.

Research questions 2, 3, and 4 examined the relationships among the study variables. Scatter plots were generated for each variable pair to identify the most appropriate model. In most cases, a linear fit line provided the best representation, indicating that linear regression models were suitable for analyzing these relationships.

To address RQ2, the impact of autism knowledge on awareness and stigma was examined using linear regression analysis in IBM SPSS. Autism knowledge was treated as the independent variable, while awareness and stigma were analyzed as separate dependent variables. The results indicated a significant positive relationship between autism knowledge and awareness (β = 0.455, t = 6.402, *p* < 0.001), with the model explaining 20.7% of the variance (R^2^ = 0.207, F = 40.981). Similarly, autism knowledge showed a significant positive relationship with stigma (β = 0.400, t = 5.461, *p* < 0.001), explaining 16.0% of the variance (R^2^ = 0.160, F = 29.823). These findings suggest that autism knowledge is associated with both awareness and stigma-related perceptions; however, the proportion of variance explained is relatively modest, indicating that these outcomes are influenced by multiple other factors beyond knowledge alone. Accordingly, while knowledge may play a role in shaping attitudes, its overall contribution to stigma appears to be partial rather than determinative, and should be interpreted within a broader set of influencing factors.

To address RQ3, linear regression analysis was conducted to examine the impact of autism misinformation on autism knowledge and awareness. Autism misinformation was treated as the independent variable, while knowledge and awareness were analyzed as separate dependent variables. The results indicated a significant positive relationship between misinformation and autism knowledge (β = 0.386, t = 5.250, *p* < 0.001), with the model explaining 14.9% of the variance (R^2^ = 0.149, F = 27.567). However, misinformation did not show a significant relationship with autism awareness (β = 0.073, t = 0.916, *p* = 0.361), and the model explained only 0.5% of the variance (R^2^ = 0.005, F = 0.839). These findings suggest that while misinformation may influence individuals’ knowledge about autism, it does not significantly affect their level of awareness. These findings also suggest that the relationship between knowledge and misinformation may be more complex than a simple inverse pattern.

To identify the primary sources of misinformation, the survey participants were asked to identify the top three sources of autism misinformation. Responses were analyzed using NVivo, and participants identified social media, websites, blogs, unverified sources, anti-vaccination platforms, and misinformed individuals—including celebrities and public figures—as major sources of misinformation. These findings highlight the need for strategies to reduce misinformation, such as labeling unreliable sources and equipping healthcare providers with resources to guide autistic individuals, family members, and caregivers toward credible information.

To address RQ4, linear regression analysis was conducted to examine the influence of cultural beliefs and social norms on autism knowledge, awareness, and misinformation. Cultural beliefs and social norms were treated as independent variables, while knowledge, awareness, and misinformation were analyzed as separate dependent variables. The results showed a statistically significant positive association between cultural beliefs and social norms and autism knowledge (β = 0.381, t = 5.15, *p* < 0.001), although the model explained a modest proportion of variance (R^2^ = 0.145, F = 26.606). A significant positive association was also observed with autism misinformation (β = 0.605, t = 9.52, *p* < 0.001), with the model explaining 36.6% of the variance (R^2^ = 0.366, F = 90.688). In contrast, no statistically significant association was found between cultural beliefs and social norms and autism awareness (β = 0.094, t = 1.18, *p* = 0.237), with very limited explanatory power (R^2^ = 0.009, F = 1.410). Overall, these findings indicate that cultural beliefs and social norms are differentially associated with autism-related outcomes, with modest explanatory power for knowledge, stronger but still limited variance explained for misinformation, and no observable relationship with awareness.

## 5. Discussion and Conclusions

The increasing prevalence of autism spectrum disorder (ASD) has intensified the need for improved understanding, early diagnosis, and supportive social environments for autistic individuals and their families. At the same time, evolving perceptions of autism, particularly those shaped by misinformation and cultural beliefs, have raised concerns about how autism is understood within society. This study examined the evolution of autism-related information in terms of autism knowledge, awareness and stigma and also explored the relationships among autism knowledge, awareness, stigma, cultural beliefs, and misinformation. Using a two-phase approach that combined a systematic literature review and a survey of autistic individuals and family members, the study aimed to better understand how these factors interact and influence perceptions of autism.

The systematic literature review revealed several important trends in the development of autism knowledge, awareness, and stigma over the past two decades. Although awareness and access to autism-related information have increased, gaps remain in several areas, particularly genetic testing knowledge and access to services. Previous studies have shown that many parents remain unaware of genetic testing options for autism or encounter barriers such as financial constraints and lack of recommendations provided. Interpretation of the genetic testing findings requires consideration of the changing healthcare and policy landscape. During earlier study periods (e.g., 2012–2013), genetic testing for autism was less routinely used, more expensive, and often less accessible because of insurance coverage and healthcare resource constraints. Broader recommendations for exome sequencing in neurodevelopmental disorders became more prominent after 2019. Therefore, reported gaps in awareness of genetic testing from studies included in the systematic review may partly reflect historical limitations in access and clinical practice rather than awareness alone. These findings highlight the need for continued education among healthcare providers, equitable access to evolving genetic services, and improved communication between professionals and families so that caregivers receive accurate, timely, and comprehensive information about current diagnostic and support resources.

The literature review also identified disparities in autism knowledge and awareness across ethnic communities. Some studies reported that certain groups, particularly Latino families, often experience lower levels of awareness and encounter barriers related to language, cultural perceptions, and access to information. These findings emphasize the importance of culturally sensitive educational initiatives and community-based outreach programs that address the unique needs of diverse populations. Additionally, the review highlighted the continued presence of autism-related stigma in many communities, which can contribute to delayed diagnoses, reduced access to services, and social isolation for families and autistic individuals. These findings suggest that while progress has been made in improving awareness, stigma and cultural misconceptions surrounding autism remain persistent challenges. Evidence from the systematic review suggested recurring patterns in autism knowledge and awareness; however, these conclusions should be considered alongside the moderate methodological quality and heterogeneity of the included studies. These findings reflect patterns reported in prior literature and should be interpreted independently from the survey findings.

The survey conducted in Phase 2 provided additional insights into perceptions of autism among autistic individuals and their family members. Overall, participants demonstrated relatively high levels of autism knowledge and moderate levels of awareness. However, variations in awareness levels were observed, indicating that not all participants possessed the same level of familiarity with autism-related information. Despite generally positive levels of knowledge, the presence of autism-related stigma was still reported among respondents, suggesting that increased knowledge alone may not necessarily eliminate negative perceptions or stereotypes associated with autism.

Qualitative responses from the survey further supported these findings. When asked to identify the primary challenges faced by autistic individuals, participants frequently referenced communication difficulties, social interaction challenges, repetitive behaviors, and socialization barriers. These responses reflect commonly recognized characteristics of autism spectrum disorder and may indicate a general or basic level of awareness of core diagnostic features rather than more detailed or nuanced understanding of the condition. Participants also identified several strategies for improving autism awareness in society, including public events, educational campaigns, and inclusive community activities. Many respondents emphasized the role of social media, workshops, and community engagement programs in fostering more supportive and informed environments for autistic individuals.

The findings related to the study’s hypotheses, derived from the survey phase data, provide additional insight into the relationships among autism knowledge, awareness, and misinformation. The results indicated a positive relationship between autism knowledge and autism awareness, suggesting that individuals who possess greater knowledge about autism are more likely to demonstrate higher levels of awareness. This finding supports the knowledge–attitude–behavior framework, which proposes that increased knowledge can contribute to greater awareness and understanding of social issues. Educational programs, public awareness campaigns, and improved access to accurate information may therefore play an important role in increasing awareness of autism and promoting more informed perceptions.

The study also examined the influence of cultural beliefs and social norms on autism-related perceptions using the survey data. The findings suggest that cultural beliefs and social norms are significantly associated with autism knowledge and autism misinformation. Cultural values and community narratives can shape how individuals interpret information about autism, influencing both the acceptance of accurate knowledge and the spread of misconceptions. In some cultural contexts, autism may be interpreted through different frameworks, which can contribute to misunderstandings about the condition. These results highlight the importance of considering cultural contexts when designing educational interventions and awareness campaigns.

One notable finding from the survey phase was the strong relationship between cultural beliefs and autism misinformation. Individuals who reported stronger adherence to cultural beliefs and social norms were more likely to endorse misinformation related to autism. This suggests that misinformation may spread more easily within social networks where cultural narratives strongly influence perceptions of health and disability. The identification of social media platforms, blogs, websites, and unverified sources as key channels for misinformation further underscores the need for improved digital literacy and the promotion of credible information sources. Healthcare providers, educators, and advocacy organizations may play an important role in guiding individuals toward reliable information and countering misinformation.

An important contribution of this study is that the findings of this study do not fully support a simple linear interpretation of the KAB model. Although autism knowledge was positively associated with awareness, increased awareness was not linked to reduced stigma. This indicates that stigma is likely shaped by a broader set of influences beyond knowledge acquisition, including emotional responses, caregiving burden, perceived severity of disability, unmet support needs, prior experiences, and prevailing social norms, suggesting that autism-related attitudes develop through more complex pathways than those outlined in traditional KAB frameworks. Notably, the survey results showed that autism awareness did not correspond to lower stigma as expected, implying that awareness and stigma may function as separate psychological constructs rather than sequential stages in a linear model. Individuals may be familiar with autism and recognize its basic features while still endorsing negative stereotypes, discomfort, or exclusionary attitudes. This aligns with wider stigma research indicating that informational exposure alone is often insufficient to shift affective or value-based judgments. It is also possible that the awareness measure reflected only superficial familiarity with autism-related terminology rather than deeper empathetic understanding or inclusive attitudes. Consequently, effective stigma-reduction strategies may need to go beyond awareness-raising and instead directly target prejudice, emotional reactions, and social norms.

One of the more unexpected findings from survey phase was the positive association between misinformation and the knowledge variable. Although this appears counterintuitive within a traditional KAB framework, several explanations are possible. First, the knowledge measure may capture general familiarity or exposure to autism-related information rather than purely objective factual accuracy. Individuals with greater exposure to autism content may encounter both accurate information and misconceptions simultaneously. Second, respondents who perceive themselves as knowledgeable may endorse some inaccurate beliefs with greater confidence. Third, the observed association may reflect limitations in item wording, scoring, or construct operationalization. Accordingly, this finding should be interpreted cautiously and highlights the need for future studies to distinguish between objective knowledge, perceived knowledge, and information exposure using more refined measures.

A notable contribution of this study from the survey phase is the identification of both online and interpersonal sources of autism misinformation. The prominence of social media and unverified online content suggests the need for stronger digital health literacy efforts, while the role of public figures and misinformed individuals indicates that misinformation may also spread through trusted social networks. These findings highlight the importance of multi-level strategies to improve access to accurate autism information.

Overall, the findings from survey phase of this study indicate that autism knowledge, awareness, cultural beliefs, and misinformation interact in complex ways. While increasing knowledge about autism can contribute to greater awareness, cultural beliefs and misinformation can shape how individuals interpret and apply that knowledge. Together, these findings suggest that autism-related perceptions are shaped by multiple interacting cognitive, social, and cultural processes. The observed relationships do not fully conform to a linear knowledge-to-attitude model, indicating that future theoretical frameworks should account for the coexistence of accurate knowledge, misinformation, cultural beliefs, and stigma. These results represent empirical findings from the present study sample. Additionally, these findings highlight the importance of comprehensive educational strategies that not only provide accurate information but also address cultural perspectives and social attitudes toward autism.

Several practical implications emerge from this research. Educational initiatives aimed at improving autism knowledge should incorporate culturally responsive approaches that recognize the diverse perspectives of different communities. Public awareness campaigns should focus on promoting accurate information about autism while actively countering misinformation circulating on social media and other online platforms. In addition, collaboration between healthcare professionals, educators, advocacy organizations, and community leaders may help ensure that autism-related information is communicated effectively and reaches a broad audience.

This study has several limitations that should be considered when interpreting the findings. First, the systematic review was restricted to U.S.-based studies to maintain consistency with Phase 2 of the research, which involved a survey of participants in the United States. Although this approach improved contextual comparability across phases, it narrowed the evidence base and may limit the generalizability of the review findings to other cultural and national contexts. Accordingly, the results should be interpreted as reflecting patterns within the included U.S.-based literature rather than global trends in autism knowledge, awareness, or stigma. Second, the literature reviewed in the systematic review phase identified several correlates of stigma, including unmet care needs, parent-reported autism severity, and caregiving demands within families. These factors may contribute to stigma independently of knowledge and awareness and are not directly addressed through informational exposure alone. This may partly explain why increased knowledge and awareness in the present study were not associated with reduced stigma. Therefore, informational interventions alone may be insufficient to reduce stigma unless they are accompanied by practical support mechanisms and family-centered services that directly address lived caregiving challenges.

Another key limitation of this study is the limited racial and ethnic diversity of the survey sample, with 84.28% of participants identifying as White. This restricts the generalizability of the findings, particularly because the systematic review identified disparities in autism knowledge, stigma, and service access among Latino and other minority communities. Consequently, the sociocultural influences observed here may not fully reflect more diverse populations. The use of Amazon Mechanical Turk (MTurk) may have contributed to this imbalance, as online convenience samples can differ from the broader population in demographic composition and access. Additionally, differential participation may also reflect sociocultural differences in autism-related perceptions and stigma. In some communities, autism may carry greater stigma or be viewed more negatively, which could reduce willingness to participate in autism-related surveys and contribute to the underrepresentation of certain racial and ethnic groups. Another limitation of this study is that the sample size was relatively modest, which may limit the statistical power of the analysis and the generalizability of the results. A limitation of this study is the restricted generalizability of the sample. Participants were recruited through MTurk and a university-affiliated autism center, and the sample was predominantly male, younger, and White. Respondents also consisted of autistic individuals, family members, and caregivers rather than the general public. Therefore, the findings reflect these stakeholder perspectives and should be generalized with caution. Another limitation of this study is the reliance on self-reported eligibility in an online survey, without external verification of participants’ connection to autism. Although eligibility criteria and targeted recruitment strategies were used, the potential for misrepresentation (“impostor” participants) cannot be entirely ruled out, which may affect the validity of the findings. Another limitation relates to measurement development. Several constructs were assessed using adapted or newly developed items, and although internal consistency reliability was acceptable, more comprehensive psychometric validation (e.g., exploratory or confirmatory factor analysis) was not feasible in the current sample. Therefore, these measures should be considered preliminary and interpreted with caution until validated in larger and more diverse samples. Also, autism is highly heterogeneous, and the current sample may not fully represent autistic individuals with higher support needs, including those with significant communication or cognitive impairments who may face barriers to participating in an online self-report survey. Consequently, the findings may more strongly reflect experiences of autistic individuals able to independently complete surveys and family respondents. Another limitation of this study is that the study’s sample combined autistic individuals and family members/caregivers, whose experiences and perceptions may differ substantially. These groups may encounter stigma through different mechanisms (self-stigma, courtesy stigma, caregiving burden, service barriers). Because the present analysis focused on the combined sample, subgroup differences were not fully examined. Future studies should analyze these populations separately. Another limitation is that autistic individuals with intellectual disability and those with higher support needs, including individuals who may meet the proposed criteria for profound autism, were likely underrepresented in the survey phase of this study. Consequently, the findings may not fully capture the experiences, perspectives, or information needs of these groups. Future studies should use more inclusive and accessible methods to better capture the perspectives of autistic individuals with intellectual disability and higher support needs. Finally, the study employed a cross-sectional design, which limits the ability to establish causal relationships between the variables examined.

Future research should expand on these findings by examining larger and more diverse populations, including participants from different cultural and geographic contexts. Longitudinal studies may also help identify how autism knowledge, awareness, and stigma evolve over time. Additionally, future studies could explore the effectiveness of targeted educational interventions designed to reduce misinformation and stigma while promoting accurate understanding of autism within diverse communities. Future research could examine whether different types of misinformation relate to stigma in different ways. In particular, it may be useful to distinguish between negative misinformation and positive but stereotyped beliefs (e.g., assuming all autistic individuals have special talents). While positive stereotypes may seem less harmful or may reduce some forms of open stigma, they can still lead to incorrect and simplified views of autism. Understanding these differences would help explain how misinformation affects attitudes and support the development of more targeted interventions. Future research using larger samples should apply structural equation modeling to more fully test the multidimensional and interrelated pathways proposed in the conceptual framework.

In conclusion, this study contributes to the growing body of research examining autism knowledge, awareness, and stigma by highlighting the complex relationships among knowledge, cultural beliefs, and misinformation. While increasing autism knowledge may enhance awareness, addressing stigma requires broader efforts that consider cultural contexts and the influence of misinformation. Educational initiatives, community engagement efforts, and improved access to reliable information may play an essential role in fostering greater understanding and more supportive environments for autistic individuals and their families.

## Figures and Tables

**Table 1:** Research Question and Search Terms.

Research Question	Search Terms
How has information about autism evolved over time, and what is the state of autism knowledge, autism awareness, and autism stigmatization in recent years?	((“autism” or “Asperger syndrome” or “asd” or “autism spectrum disorder” or “autistic disorder” or “pervasive developmental disorder”) AND (“knowledge” or “awareness” or “stigma”) AND (“autistic patients” or “autistic individuals” or “autistic” or “family” or “caregivers”))

**Table 2:** Quality Assessment of Included Studies (N = 23).

Study	Design	Sample Size	Data Collection	Risk of Bias	Quality
Sansosti et al. (2012)	Qualitative	16	Interviews	High (small sample)	Low
Vande Wydeven et al. (2012)	Survey	155	Questionnaire	Moderate	Moderate
Chen et al. (2013)	Qualitative	42	Interviews	Moderate	Moderate
Magana et al. (2013)	Survey	104	Questionnaire	Moderate	Moderate
Zuckerman et al. (2014)	Qualitative	33	Focus groups/interviews	Moderate	Moderate
Blanche et al. (2015)	Qualitative	15	Interviews	High	Low
Jones et al. (2015)	Qualitative	10	Interviews	High	Low
Deyro et al. (2016)	Survey	83	Questionnaire	Moderate	Moderate
Ijalba (2016)	Qualitative	22	Interviews	High	Low
Ratto et al. (2016)	Survey	56	Questionnaire	Moderate	Moderate
Burke et al. (2018)	Survey	99	Questionnaire	Moderate	Moderate
Cohen and Miguel (2018)	Qualitative	25	Interviews	High	Low
Dababnah et al. (2018)	Qualitative	22	Interviews	High	Low
Hanish et al. (2018)	Qualitative	20	Interviews	High	Low
Harrington et al. (2018)	Survey	143	Questionnaire	Moderate	Moderate
Marsack and Perry (2018)	Qualitative	51	Interviews	Moderate	Moderate
Zuckerman et al. (2018)	Survey	380	Questionnaire	Low	High
Ferguson and Vigil (2019)	Survey	47	Questionnaire	Moderate	Moderate
Gordillo et al. (2020)	Qualitative	20	Interviews	High	Low
Habayeb et al. (2020)	Qualitative	20	Questionnaire and follow-up phone interviews	High	Low
Zebolsky et al. (2020)	Survey	138	Questionnaire	Moderate	Moderate
Rosales et al. (2021)	Qualitative	28	Interviews	High	Low
Stein Duker et al. (2022)	Qualitative	31	Interviews	Moderate	Moderate

**Table 3:** Cronbach Alpha for Each Autism Variable.

Variables	Cronbach Alpha
Autism Knowledge	0.773
Autism Awareness	0.763
Autism Stigma	0.706
Autism Cultural Beliefs and Social Norms	0.942
Autism Misinformation	0.957

**Table 4:** Descriptive Statistics (N = 159).

Variable	N	Minimum	Maximum	Mean	Std. Deviation
Knowledge	159	1.6	4.8	3.811	0.5177
Awareness	159	2.8	5.0	3.981	0.4588
Stigma	159	1.0	4.8	3.737	0.6228
Cultural Beliefs and Social Norms	159	1.0	5.0	3.629	0.7204
Misinformation	159	1.6	5.0	3.517	0.6944

**Table 5:** Sociodemographic Characteristics of Participants (N = 159).

Socio-Demographics	Characteristics	N	%
Gender	Male	105	66.04
	Female	54	33.96
Age	18–30 years	74	46.54
	31–40 years	60	37.74
	41–50 years	16	10.06
	51–60 years	6	3.77
	61–70 years	3	1.89
Ethnicity	White	134	84.28
	Black or African American	1	0.63
	American Indian or Alaska Native	3	1.89
	Asian	20	12.58
	Native Hawaiian or Pacific Islander	0	0.00
	Other	1	0.63

**Table 6:** Background Question Responses of Participants (N = 159).

Questions	Responses	n	%
Do you know someone with autism	Yes	159	100
	No	0	0
If you know someone with autism, then what is your relationship with them?	Self	62	38.99
	Family Member	97	61.01
	Other	0	0
Please rate your understanding of autism.	Very familiar with autism	35	22.01
	Some understanding/Familiar with autism	95	59.75
	Minimal/Very little understanding	29	18.24
For how many years have you been familiar with the concept of autism?	1–10 years	124	77.99
	11–20 years	24	15.09
	20+ years	11	6.92
What is your primary/main source of information for your knowledge about autism?	Personal Experiences	72	45.28
	Doctor or other medical professional	61	38.36
	Learned about autism at school/university	13	8.18
	Internet	10	6.29
	News	3	1.89
	Social media.	0	0.00
	TV shows/movies	0	0.00
	Research Articles	0	0.00
	Others	0	0.00
What is your secondary source of information for your knowledge about autism?	Doctor or other medical professional	46	28.93
	Personal Experiences	41	25.79
	Internet	29	18.24
	Learned about autism at school/university	27	16.98
	TV shows/movies	9	5.66
	News	4	2.52
	Social media	1	0.63
	Research Articles	1	0.63
	Others	1	0.63

**Table 7:** Andrew F Hayes Mediation Process Analysis Results.

Predictor	B	SE	t	*p*	95% CI
Knowledge → Stigma	0.491	0.099	4.959	<0.001	[0.296, 0.688]
Awareness → Stigma	−0.027	0.112	−0.243	0.809	[−0.248, 0.194]

## Data Availability

Data is not publicly available due to ethical restrictions but will be made available on request.

## Supplementary Materials

The following supporting information can be downloaded at: https://www.mdpi.com/article/10.3390/bs16050660/s1.
