# Supplementary material for: Autism Information Progression and the Impact of Misinformation on Autism Knowledge, Awareness and Stigmatization
Source: Behav Sci (Basel). 2026 Apr 27;16(5):660. doi: 10.3390/bs16050660 (PMC13203535; doi:10.3390/bs16050660)
Supplement: Supplementary file 1 [file behavsci-16-00660-s001.zip › behavsci-4231559-supplementary.pdf]

SURVEY

QUESTIONNAIRE

B.1. Demographics Questions

1. Age
   ____

2. Gender
   - o Male
   - o Female
   - o Non-binary / third gender
   - o Prefer not to answer.

3. Ethnicity
   - o White
   - o Black or African American
   - o American Indian or Alaska Native
   - o Asian
   - o Native Hawaiian or Pacific Islander
   - o Other

B.2. Background Questions

4. Do you know someone with autism?
   - o Yes
   - o No

5. If you do know someone with autism, then what is your relationship with them?
   - o Self
   - o Family member. Please mention the relationship: _____
   - o Other. Please mention the relationship: _____

6. Please rate your understanding of autism.
   - o Minimal/Very little understanding
   - o Some understanding/Familiar with autism
   - o Very familiar with autism

7. For how many years have you been familiar with the concept of autism?
   ____

8. What is your primary/main source of information for your knowledge about autism? (Choose all that may apply)

- o Personal experiences
- o Doctor or other medical professional
- o Learned about autism at school/university
- o TV shows/movies
- o Internet
- o News
- o Social media. Please specify the media name. _____
- o Research articles.
- o Other. Please specify: _____

9. What is your secondary source of information for your knowledge about autism? (Choose all that may apply)
   - o Personal experiences
   - o Doctor or other medical professional
   - o Learned about autism at school/university
   - o TV shows/movies
   - o Internet
   - o News
   - o Social media. Please specify the media name. _____
   - o Research articles.
   - o Other. Please specify: _____

10. What are the three significant difficulties or obstacles you believe individuals with autism might encounter?

    ______

B.3. Autism Knowledge Questions

11. Are you aware that the prevalence of autism spectrum disorder (ASD) is such that around 1 in 36 children has been diagnosed with it?
    - o Yes
    - o No

12. As of now, autism is a condition that cannot be cured.
    - o Yes
    - o No

13. Attention difficulties are frequently found in children with ASD.
    - o Yes
    - o No

14. Please rate how much you agree or disagree with each of the following statements?

	Strongly disagree	Somewhat disagree	Neither agree nor disagree	Somewhat agree	Strongly agree
a. Clinical observation is the best way to diagnose autism.	○	○	○	○	○
b. Early intervention can lead to significant gains in children with autism's social and communication skills.	○	○	○	○	○
c. Autism is more frequently diagnosed in males than in females.	○	○	○	○	○
d. Autism can be diagnosed as early as 18 months.	○	○	○	○	○
e. Autism does not exist only in childhood.	○	○	○	○	○

B.4. Autism Awareness Questions

15. Could you please list what you perceive as the three effective ways to increase autism awareness among individuals and the society?

    ____

16. Did you know that children with autism may have strange reactions to the way things smell, taste, look, feel, or sound.
    - o Yes
    - o No

17. Do you agree that people with autism are generally very good in attributing intentions, desires, and beliefs to others.
    - o Yes
    - o No

18. Please rate how much you agree or disagree with each of the following statements?

	Strongly disagree	Somewhat disagree	Neither agree nor disagree	Somewhat agree	Strongly agree
a. Many children with autism get upset if their routine is changed.	○	○	○	○	○
b. Some children with autism show intense interest in parts of objects.	○	○	○	○	○
c. The symptoms of ASD are very different from one person to another.	○	○	○	○	○
d. Behavior therapy is an intervention most likely to be effective for children with autism.	○	○	○	○	○
e. Many children with autism have difficulty using everyday language to communicate their needs.	○	○	○	○	○

B.5. Autism Stigma Questions

19. Autism is considered as a stigma and is impacting the wellbeing of autistic people.
   - Yes
   - No

20. Autism is preventable.
   - Yes
   - No

21. Do you agree that autism happens mostly in middle class families.
   - Yes
   - No

22. Please rate how much you agree or disagree with each of the following statements?

	Strongly disagree	Somewhat disagree	Neither agree nor disagree	Somewhat agree	Strongly agree
a. Autism is due to cold, rejecting parents or due to traumatic experiences.	○	○	○	○	○
b. Autistic children usually grow up to be schizophrenic adults.	○	○	○	○	○
c. Most children with autism are extremely impaired and cannot live independently as adults.	○	○	○	○	○
d. Most children with autism are also intellectually disabled.	○	○	○	○	○
e. There is a negative opinion towards children diagnosed with autism in some communities.	○	○	○	○	○

B.6. Autism Cultural Beliefs and Social Norms Questions

23. Autism is a mental illness.
    - o Yes
    - o No

24. It is important that all children diagnosed with autism receive some form of special education services at school.
    - o Yes
    - o No

25. Autism is caused by God or a supreme being.
    - o Yes
    - o No

26. Please rate how much you agree or disagree with each of the following statements?

	Strongly disagree	Somewhat disagree	Neither agree nor disagree	Somewhat agree	Strongly agree
a. Autism is a result of a curse or evil eye put upon/inflicted on the family.	○	○	○	○	○
b. Autism tends to run in families and can be inherited.	○	○	○	○	○
c. There are cultural methods and support that are effective in helping people with autism.	○	○	○	○	○
d. Autism is something caused by environment.	○	○	○	○	○
e. Autism affects people of with certain races and ethnicities.	○	○	○	○	○

B.7. Autism Misinformation Questions

27. Autistic children are deliberately negativistic and non-complaint.
    - o Yes
    - o No

28. Do you agree that we now have treatments that can cure autism.
    - o Yes
    - o No

29. Do you agree that a child can grow out of autism.
    - o Yes
    - o No

30. Please list three information sources that you believe are responsible for spreading misinformation about autism.

    ____

31. Please rate how much you agree or disagree with each of the following statements?

	Strongly disagree	Somewhat disagree	Neither agree nor disagree	Somewhat agree	Strongly agree
a. Autism is caused by vaccines.	○	○	○	○	○
b. Autism is a communication disorder.	○	○	○	○	○
c. Autism occurs more commonly among higher socio- economic and educational levels.	○	○	○	○	○
d. Autistic children do not show social attachments, even to parents.	○	○	○	○	○
e. Autism is a genetic disease.	○	○	○	○	○
